# MONICEPH project: Monitoring cephalopods during whale-watching activity in the Azores (2020-2024)

**DOI:** 10.3897/BDJ.13.e158822

**Published:** 2025-10-20

**Authors:** Stéphanie R.A. Suciu, Jean-Luc Jung, José M.N. Azevedo

**Affiliations:** 1 Institute of Marine Sciences - OKEANOS, University of the Azores, 9500-321 Ponta Delgada, Portugal Institute of Marine Sciences - OKEANOS, University of the Azores 9500-321 Ponta Delgada Portugal; 2 Institut de Systématique, Évolution, Biodiversité (ISYEB), Muséum National d’Histoire Naturelle, CNRS, Sorbonne Université, EPHE-PSL, Université des Antilles, 75005 Paris, France Institut de Systématique, Évolution, Biodiversité (ISYEB), Muséum National d’Histoire Naturelle, CNRS, Sorbonne Université, EPHE-PSL, Université des Antilles 75005 Paris France; 3 Station Marine de Dinard du Muséum National d’Histoire Naturelle, 35800 Dinard, France Station Marine de Dinard du Muséum National d’Histoire Naturelle 35800 Dinard France

**Keywords:** citizen science, ocean, opportunistic data, whale-watching

## Abstract

**Background:**

The study of oceanic cephalopods off the Azores Archipelago began decades ago with the analysis of stomach contents from sperm whales that were hunted for the whaling industry. The identification of numerous cephalopod species contributed significantly to cephalopod taxonomy, as well as enhancing understanding of the sperm whale diet. In the 1990s, the shift from whaling to whale-watching created new opportunities to continue studying deep-ocean ecology: participatory research involving the actors of the new industry.

**New information:**

MONICEPH (MONItoring CEPHalopods during whale-watching activity in the Azores) is a collaborative platform designed to collect, organise and disseminate cephalopod occurrence data gathered by whale-watching companies in the Azores. From 2020 to 2024, cephalopod remains found at the water's surface during sightings of cetaceans were collected in partnership with companies from four islands: São Miguel, Terceira, Pico and Faial. The deep-ocean cephalopod remains at the water’s surface were likely brought up by their predators during feeding activity. We assume that sperm whales, in particular, occasionally release cephalopods at the surface due to incomplete consumption during a hunt or for feeding of their calves. Trained staff collected the samples, which were subsequently identified using DNA barcoding and/or morphological characteristics. The dataset includes 182 cephalopod records across 16 species. One species, *Onykia carriboea* Lesueur, 182, has been newly identified in the region, expanding the list of species previously documented in the published data for the Northeast Atlantic.

## Introduction

**Importance of cephalopods**

Deep-ocean cephalopods have a structural role in the oceanic ecosystem: supporting trophic interactions, linking lower levels (such as micronekton) to apex predators ([Bibr B12913030][Bibr B12913116], Golikov et al. 2019, Escánez et al. 2021); facilitating nutrient cycling through vertical migrations; or allowing carbon sequestration to the ocean floor (Rosa et al. 2013, de la Chesnais et al. 2019). Moreover, due to their short life span and their sensitivity to environmental changes such as temperature and oxygen levels, cephalopod populations can serve as indicators of ocean health (Jackson and Moltschaniwskyj 2002, Forsythe 2004), as fluctuations in their occurrence and abundance can provide insights into the impacts of climate change on marine ecosystems (Boavida-Portugal et al. 2022).

**The challenge of direct sampling, particularly for deep sea cephalopods**

The large variety in cephalopod morphology, habitat and life-history strategies hampers the use of a single standardised sampling method. Direct sampling methods include highly invasive trawling or video surveys (Hoving et al. 2014). However, in addition to being expensive and, therefore, limited in time and space, each of these methods has its own biases. The sophisticated features associated with the predatory behaviour of cephalopods, such as vision and agility, for example, may result in avoidance behaviour towards many types of oceanographic gear (Villanueva et al. 2017). On the other hand, specimens captured in trawled nets may be damaged, making their morphological identification challenging (Vecchione et al. 2010).

**Indirect methods to study deep sea cephalopods**

Stomach content analysis of teuthophagous cetaceans has been an indirect method to study deep-ocean cephalopods on commercially hunted (Clarke et al. 1993) or stranded (e.g. Foskolos et al. (2020)) animals. Being crucial to looking at cetacean diet, this method has limitations for studying cephalopod ecology, stemming from predator selectivity and imprecise location data. Even so, it has been claimed that stomach content analysis provides a better overview of cephalopod distribution and relative abundance than net catches (Clarke 2006). Some of the methodological, logistic and ethical limitations to sampling pelagic cephalopods can nevertheless be overcome with molecular approaches (O'Brien et al. 2018).

Modern molecular tools allow indirect methods of studying cephalopods and determining their presence in a region. Analysis of environmental DNA (DNA fragments persisting in environmental samples) has offered non-invasive methods to study the community composition (Valentini et al. 2009, Berry et al. 2017, Ford et al. 2016). At the individual level, DNA barcoding allows species identification based on an organism’s remains (Hoving et al. 2014, Xavier et al. 2015). The high costs of using sophisticated technological means to access deep-sea ecosystems give an inestimable value to specimens collected in these environments (Sampaio et al. 2019).

**From whaling to whale-watching: an opportunity for participatory science**

The Azores Archipelago, with its diverse submarine topography, is a key area for marine biodiversity, including cephalopods, which serve as primary prey for several cetacean species. From the nineteenth century, sperm whales have been extensively hunted in the region. Malcolm R. Clarke (1930–2013), a British zoologist who settled on Pico Island, dedicated most of his career to studying sperm whales and their prey, the cephalopods. His work on cephalopod beaks recovered from sperm whale stomachs provided valuable insights into oceanic ecosystems. Clarke's research led to the identification of numerous cephalopod species, significantly advanced cephalopod taxonomy and provided valuable insights into sperm whales' diet (Clarke 1996b, Gomes-Pereira et al. 2014). This kind of research was halted following the International Whaling Commission's 1980s moratorium on whaling. In the 1990s, the shift from whaling to whale watching created new opportunities to continue studying deep-ocean ecology. This transition enabled a participatory research approach, involving actors in the new industry who, through their daily experiences at sea, were able to observe and report ecological situations/phenomena that traditional research alone could not capture. This approach provides a cost-effective method for surveying and gathering data that would otherwise be difficult to obtain (Coché et al. 2021, González García et al. 2023). It can also complement traditional surveys, especially in areas lacking baseline data or where funding is scarce or unavailable.

## General description

### Purpose

This publication presents the MONICEPH dataset of oceanic cephalopod occurrences, derived from opportunistic samples collected by whale-watching companies in the Azores. The dataset has been made publicly available through Global Biodiversity Information Facility (GBIF), Ocean Biodiversity Information System (OBIS) and European Marine Observation and Data Network (EMODNET) in the form of a Darwin Core Archive (DwC-A). The links to the dataset are available on the dataset dedicated page of the Flanders Marine Institute (VLIZ).

Our objectives are to: (1) provide a comprehensive description of the data collection methodology, (2) suggest possible uses for the dataset and (3) encourage the replication of this initiative in other areas.

## Project description

### Title

MONICEPH: Monitoring cephalopods during whale-watching activities in the Azores

### Personnel

Project coordination was done by Stéphanie R.A. Suciu and José M. N. Azevedo. Data presented here have been collected by people working for 13 whale-watching companies operating on four islands: Faial (Azores Experiences, Dive Azores, and Norberto Diver), Pico (Aqua Açores, CW Azores, Espaço Talassa and Futurismo), São Miguel (Futurismo, Moby Dick, Picos de Aventura, Terra Azul, Terra do Pico and Sea Colors) and Terceira (Water4Fun) and Futurismo Azores Adventures in Pico and in São Miguel. Additional samples were received from four research projects or organisations: the Delphis project, ElasmoBase, Whale Marine Conservation Research International and Nova Atlantis.

### Study area description

**Figure 1.**
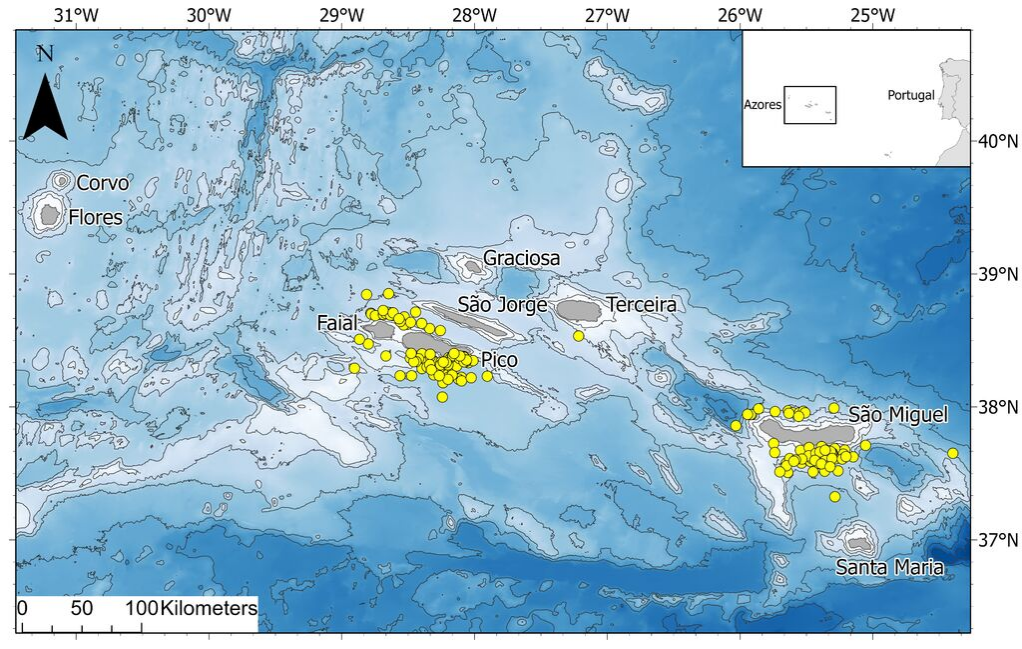
The Azores Archipelago, with the locations (yellow dots) of the samples compiled on the MONICEPH database (except the Architeuthis dux sample collected during the Marine Conservation Research International expedition of 2019, approximately 1000 km south of the Azores).

Maritime area surrounding the Azores Archipelago (Fig. 1)

The Azores, an archipelago in the North Atlantic Ocean, is characterised by its unique geography and oceanographic conditions, creating an ideal environment for cephalopods (Santos et al. 1995, Clarke 1996a, Morato et al. 2020). Located at the intersection of three tectonic plates, its complex bathymetry (including seamounts, abyssal plains and steep underwater slopes) creates an ideal habitat for deep-sea cephalopods, which thrive in mesopelagic to bathypelagic zones (Caldeira and Reis 2017). Moreover, the oceanographic dynamic, driven by its position at the convergence of major ocean currents (the Gulf Stream, the Canary Current and the Azores Current), creates eddies and upwelling zones, transporting nutrients and supporting a highly productive ecosystem (Sala et al. 2021, Frazão et al. 2022). Finally, the seafloor around the Azores is shaped by volcanic activity and hydrothermal vents, fostering deep-sea habitats (Colaço et al. 2006, Couto et al. 2015).

### Design description

MONICEPH is a collaborative platform to encourage and facilitate the collection of oceanic cephalopod samples in the Azores. It strives to identify the species from pictures, from morphological analysis of the samples and from DNA (meta)barcoding. All data are open access, and samples are made available for further studies. It works mostly with whale-watching companies, but welcomes samples from any source.

### Funding

The costs of the project have been supported by Projects Portal da Biodiversidade dos Açores (2022–2023) – PO Azores Project - M1.1.A/INFRAEST CIENT/001/2022; FCT-UIDB/00329/2020-2024, DOI 10.54499/UIDB/00329/2020 (Thematic Line 1 – integrated ecological assessment of environmental change on biodiversity); DRCT Pluriannual Funding (M1.1.A/FUNC.UI&D/010/2021-2024) to the Island Biodiversity, Biogeography & Conservation (IBBC) group within the Centre for Ecology, Evolution and Environmental Changes (CE3C); and M1.1.C/PROJ. EXPLORATÓRIOS/013/2022 grant from the Regional Direction for Science and Technology (DRCT) – Açores (Portugal). This work received national funds through the FCT – Foundation for Science and Technology, I.P., under the projects UIDB/05634/2020 and UIDP/05634/2020 and through the Regional Government of the Azores through the project M1.1.A/FUNC.UI&D/003/2021-2024. The Boost 30+ grant, the Mobility programme from Actiris Brussels (Belgium) (2020) and the M3.1.a/F/003/2021 grant from the Regional Science and Technology Fund (FRCT), Ponta Delgada – Açores (Portugal) (2021–2024), covered the human resource fees for Stéphanie R.A. Suciu.

## Sampling methods

### Study extent

This study focuses on the waters of the Azores, mostly on the areas around the islands of São Miguel, Terceira, Pico and Faial used by whale-watching boats (Fig. 1). Data were collected primarily during the summer months when sea conditions are optimal and tourism activity is at its peak.

### Sampling description

A total of 13 whale-watching companies participated. Each company received a boxed sampling kit and the staff were trained in sampling and observations as described in Suciu et al. (2021). Every time cephalopod remains were observed, a sample was taken and preserved in 96% ethanol in supplied jars. Additionally, the collectors recorded, as a minimum, the time and geographic position of the finding and information about the species of cetaceans in the area and their behaviour. When possible, photographs of the remains were also taken. Upon return to land, the samples were stored at 4°C in a fridge.

### Step description

The data have been published in the standardised format Darwin Core Archive (DwC-A) for biodiversity data. It consists of a core data file (occurrence table) that contains information about the location and time of the sampling, associated with the taxonomical information; the occurrenceID field links with an extension data table, the DNAderivatedTable, which contains information for the DNA barcoding identification (i.e. primers) and the sequence of the amplified COI fragment (DNA sequence).

**Species identification**: When available, morphological observations and/or collected images were analysed to determine species using the identification keys of Gomes-Pereira et al. (2016). For a large majority of samples, DNA barcoding analysis was performed.

DNA extraction was performed with the E.Z.N.A.® Mollusc DNA Kit (Omega Bio-tek, Georgia, USA), according to the manufacturer’s protocol. Two primers, HCO2198 and LCO1490 (Folmer et al. 1994), were used to PCR amplify a partial 710-bp fragment from the mitochondrial COI gene using the conditions in Folmer et al. (1994). The amplicons generated were individually Sanger-sequenced with BigDye Terminator 3.1, then run on ABI 3730XL (Applied Biosystems, Foster City, CA, USA), using the forward PCR primer. Forward sequences were cleaned and reviewed manually (inspection of the chromatograms) using Geneious (v. 2024.0.7, Biomatters, Auckland, NZ), then compared to the NCBI nucleotide database and the BOLD database using the BLAST algorithm for taxonomic assignment.

Identity results of the BLAST for each sample and comparison between databases are shown in Table 1

## Geographic coverage

### Description

Archipelago of the Azores

### Coordinates

36.712 and 39.91 Latitude; −31.575 and −24.5 Longitude.

## Taxonomic coverage

### Description

There is a total of 16 species of cephalopods occurring in the dataset, from 11 different families and two orders. For one sample, the identification was done at the genus level, with an ambiguity between two taxa at the species level (*Stigmatoteuthis arcturi* or *Stigmatoteuthis hoylei*). The orders, species and families and the number of records per taxa are covered in Table 2.

## Temporal coverage

**Data range:** 2019-6-09 – 2024-9-20.

### Notes

Most samples were collected in summer.

## Collection data

### Collection name

A total of 182 cephalopod records have been included in MONICEPH from 2019 to 2024. A sample was collected for 176 of them. The samples are stored in ethanol and housed at the Faculty of Sciences and Technology of the University of the Azores, in Ponta Delgada.

## Usage licence

### Usage licence

Other

### IP rights notes

Creative Commons Attribution 4.0 Internacional

## Data resources

### Data package title

MONICEPH - Monitoring cephalopods during whale-watching activity in the Azores

### Resource link

https://doi.org/10.15468/ebu243

### Alternative identifiers

https://vliz.be/en/imis?module=dataset&dasid=8748

### Number of data sets

2

### Data set 1.

#### Data set name

Occurrence

#### Description

The dataset was published in the Global Biodiversity Information Facility platform, GBIF (Suciu and Azevedo 2025). The core data file contains 182 records with the following variables:.

**Data set 1.** 

Column label	Column description
occurrenceID	Unique identifier of the observation.
modified	The date of the last modification of the resource.
collectionCode	Abbreviation of the dataset name, in this case, MONICEPH.
basisOfRecord	The specific nature of the data record, in this case, HumanObservation or MaterialSample.
decimalLatitude	The latitude (in decimal degrees, using the spatial reference system in geodeticDatum) of the location of the encounter.
decimalLongitude	The longitude (in decimal degrees, using the spatial reference system in geodeticDatum) of the location of the encounter.
maximumDepthInMetres	The maximum depth at which the animal was found (always 0 m, as they are recorded at the surface).
minimumDepthInMetres	The minimum depth at which the animal was found (always 0 m, as they are recorded at the surface).
coordinateUncertaintyInMetres	The horizontal distance from the given dwc:decimalLatitude and dwc:decimalLongitude describing the circle with the occurrence location.
countryCode	Standard code of the location, in this case “PT” for Portugal.
islandGroup	The island group of the occurrence, in this case: “Açores, Arquipélago dos”.
island	Name of the nearest island of the occurrence.
eventDate	Date when an occurrence was registered. Formatted as 'YYYY-MM-DD’.
eventTime	Time of the occurrence. Formatted as ‘HH:MM-0100’ in the Azores time, UTC-1.
scientificName	Complete scientific name including author and year.
scientificNameID	Life Sciences Identifier (LSID) assigned to the taxon by WoRMS.
scientificNameAuthorship	Name of the author of the lowest taxon rank included in the record.
taxonRank	Lowest taxonomic rank of the record.
kingdom	Kingdom name.
class	Class name.
order	Order name.
superfamily	Superfamily name.
family	Family name.
subfamily	Subfamily name.
genus	Genus name.
licence	The licence describing how the resource can be used.
rightsHolder	The person managing the resource.
individualCount	The number of individuals present at the time of the dwc:Occurrence.
institutionCode	The identity of the institution publishing the data.
recordedBy	The person responsible for recording the original dwc:Occurrence.
occurrenceStatus	Statement on presence or absence, in this case "present".
samplingProtocol	The method used during a dwc:Occurence “Observation of cephalopods opportunistically found at the sea surface” or “remains of cephalopods opportunistically found at the sea surface and preserved in 96% ethanol”.
identificationRemarks	Allow to give identification details for specimen with different possible taxa.
identifiedBy	The person who assigned the dwc:Taxon to the subject.
identificationReferences	The reference used to identify the taxon.
associatedSequences	The genetic sequence information associated with the dwc:MaterialSample.
associatedMedia	Media (photos) taken by the sample collector, associate with the dwc:Occurrence.
occurrenceRemarks	Notes about the dwc:Occurrence (generally about the appearance of the cephalopod remains and/or about the cetaceans present in the area).
datasetName	The name identifying the dataset, in this case,"MONICEPH - Monitoring cephalopods during whale watching activity in the Azores".
vitality	An indication of whether the organism associated with the dwc:occurence was alive or dead at the time of collection or observation.
associatedTaxa	Taxa of cetaceans observed in the area of the cephalopod sample, not necessarly a predator.
language	language of the resource, in this case, English and Portuguese.
catalogNumber	the unique identifier for the record within the MONICEPH collection.
country	country in which the dcterms:island occurs, in this case, Portugal.

### Data set 2.

#### Data set name

DNA-derivated data

#### Description

The DNA derivated table contains 118 DNA enriched entries (DNA sequences).

**Data set 2.** 

Column label	Column description
occurrenceID	The occurrence ID allowing to link the additional table (DNA derivated data) to the core table (occurrence table).
DNA_sequence	The DNA sequence that allowed the taxonomic interpretation.
ampliconSize	The length of the amplicon in base-pairs.
target_gene	Targeted gene in the PCR (in this case, the mitochondrial cytochrome c oxidase subunit I gene (COI).
target_subfragment	Name of subfragment of a gene.
pcr_primer_forward	Sequence of the forward PCR primer that was used to amplify the sequence of the targeted gene.
pcr_primer_reverse	Sequence of the reverse PCR primer that was used to amplify the sequence of the targeted gene.
pcr_primer_name_forward	Name of the forward PCR primer (in this case: LCO1490).
pcr_primer_name_reverse	Name of the reverse PCR primer (in this case: HCO2198).
seq_meth	Sequencing method used.
seq_quality_check	Indicate if the sequence has been reviewed automatically or manually (in this case, all sequences have undergone a manual editing procedure.
pcr_primer_reference	Reference for the primers.

## Additional information

This participatory science initiative is highly replicable and can be easily adapted to other regions. Through this study, we aim to demonstrate that the implementation of such a programme not only aids in updating regional species lists, but also enhances our understanding of predator-prey interactions, thereby contributing to a deeper knowledge of local biodiversity. We present a brief analysis of the dataset to illustrate the scientific potential of participatory research with whale-watching companies.

**Figure 2.**
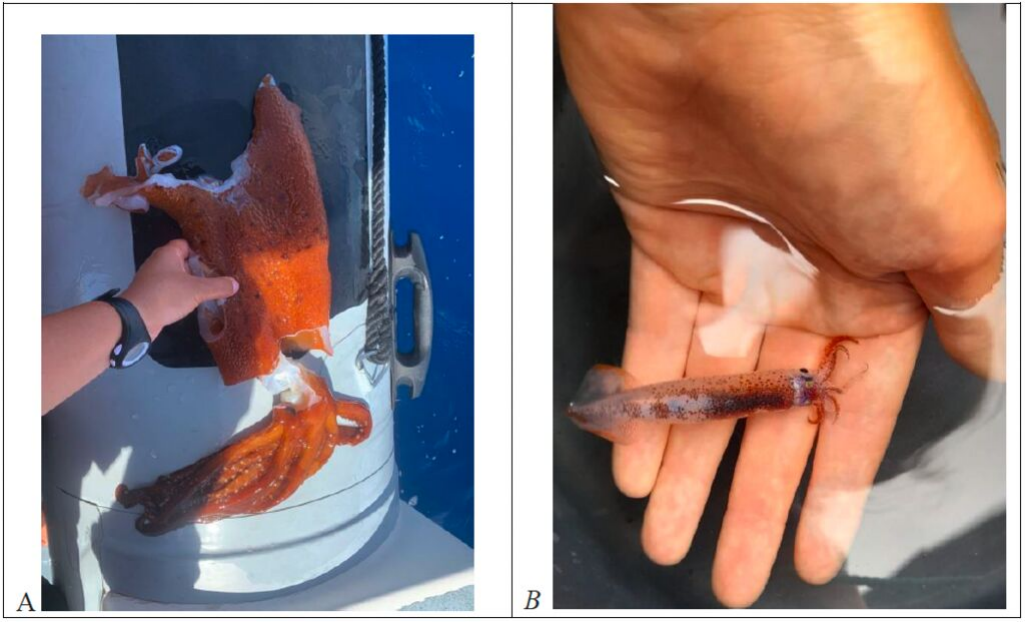
(A) New oceanic cephalopod recorded for the Azores, *Onykia carriboe*, collected off Pico Island by Isaure Michaud with Espaço Talassa. Clear signs of predation are visible. It was found in an area where short-finned pilot whales (*Globicephala macrorhynchus*) were sighted. (B) *Ommastrephes caroli* collected off Pico Island by Arianna Fornaroli with Espaço Talassa. This specimen jumped inside the boat earlier and was found dead.

This study has allowed us to update the list of species present in the region with the new record of *Onykia carriboe* Lesueur, 1821 (Fig. 2A) to the Azores cephalopod fauna: this species is not included on recent checklists (review in prep. by the authors) or online biodiversity databases.

Our dataset contains mostly deep-ocean cephalopods, which we assume is evidence that they have been released at the surface by cetaceans as a result of regurgitation or incomplete consumption. The only exception was the neon flying squid, which jumped inside the boat (Fig. 2B). The identification of cetacean species was conducted by the guide or biologist aboard the whale-watching boat.

The occurrenceRemarks and associatedTaxa fields of the dataset are used to record the cetacean species present in the area where the cephalopod was found. This information is summarised in Table 3. In 20 samples, this field is empty, but in 12 others, there is the specific note that no cetaceans were sighted in the area. More than half of the associations involved sperm whales. In one quarter of those, a calf was present. In one specific instance, Lynn Kulike at Espaço Talassa recorded that a specimen of *Stigmatoteuthis arcturi* was regurgitated by a female adult sperm whale to the calf by her side, the sample having a strong smell of stomach contents when a piece was collected.

**Figure 3.**
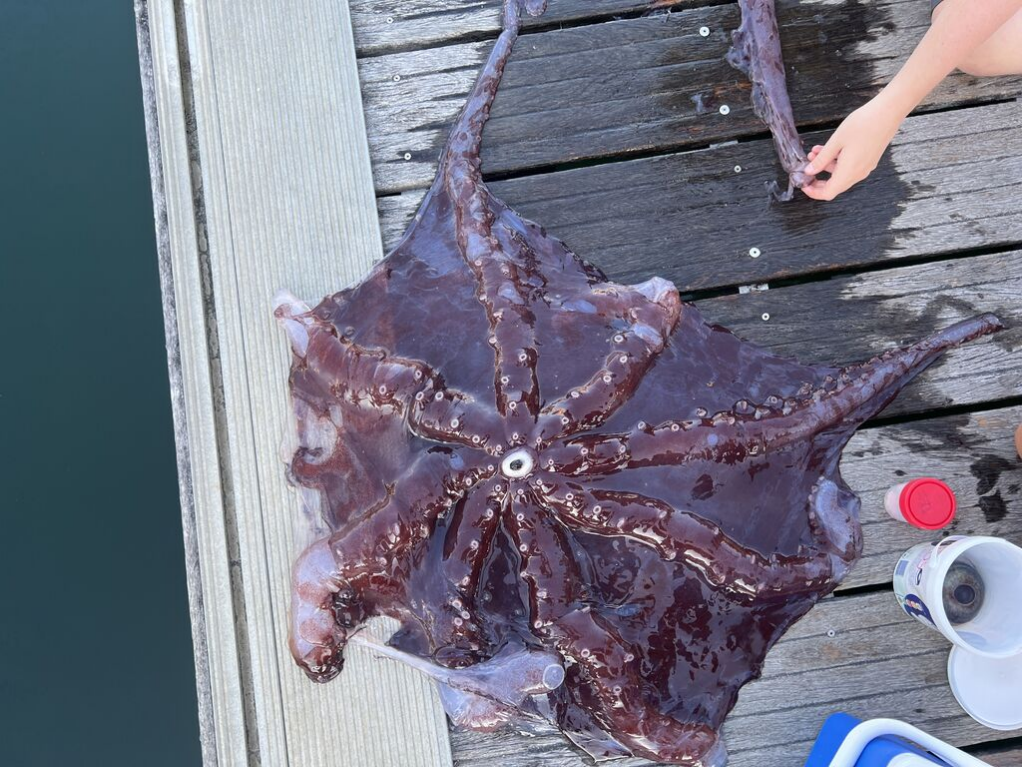
Remains of *Haliphron atlanticus* (sample 2024SMG186, collected by Terra Azul).

Most of the samples associated with sperm whales (indeed, over 90% of all records) involve *Haliphron atlanticus*. This gelatinous octopus (Fig. 3) is found in meso- and benthopelagic environments, typically associated with continental slopes (Miller et al. 2018). The high frequency of observations does not necessarily reflect cetacean feeding preferences. Observations from various regions, especially those involving foraging sperm whales, suggest that bringing this large octopus to the surface may serve purposes beyond feeding, such as helping to teach calves how to hunt or echolocate prey (Suciu et al. 2021).

Another interesting observation was made with short-finned pilot whales (*Globicephala macrorhynchus*): for a particular pod on a specific day, in addition to samples of *Todarodes sagittatus* found at the surface, soft remains of this species were found directly in the faeces, identified with DNA barcoding.

Finally, in some cases, samples were found during observation of non-teuthophagous cetaceans, alone or in association with teuthophagous cetaceans.

As several species of cetaceans can occupy the same area and some may be overlooked due to the opportunistic nature of the work, linking cetaceans to the presence of cephalopods is challenging. A future upgrade of the MONICEPH programme could incorporate DNA detection of the predator on the cephalopod samples, which would enhance our understanding of prey-predator interactions. Additionally, tools such as environmental DNA (eDNA), could complement the cephalopod monitoring programme, allowing for comparisons of results and an evaluation of how the MONICEPH programme can serve as a proxy for cephalopod occurrence during whale-watching activities in the region.

Despite their pivotal role in the world oceans and their role as indicators of the marine environment, data on cephalopod distribution are lacking for most species that inhabit the deep sea. By collecting ecological data over a wide oceanic area and across multiple years, we are encouraging this study to be continued in the long term and for similar projects to be replicated in other regions.

## Figures and Tables

**Table 1.** BLAST taxonomic assignment results in the NCBI nucleotide database and the BOLD database.

catalogNumber	NCBI	%identity (NCBI)	Accession Number (NCBI)	BOLD	%identity (BOLD)
2019SMG1	*Haliphron atlanticus*	99.85	AY557516.1	*Haliphron atlanticus*	100.00
2019SMG2	*Haliphron atlanticus*	100.00	AY557516.1	*Haliphron atlanticus*	100.00
2019SMG4	*Haliphron atlanticus*	100.00	AY557516.1	*Haliphron atlanticus*	100.00
2020SMG5	*Haliphron atlanticus*	100.00	AY557516.1	*Haliphron atlanticus*	100.00
2020SMG7	*Haliphron atlanticus*	100.00	AY557516.1	*Haliphron atlanticus*	100.00
2020SMG8	*Haliphron atlanticus*	99.85	AY557516.1	*Haliphron atlanticus*	100.00
2020SMG9	*Haliphron atlanticus*	100.00	AY557516.1	*Haliphron atlanticus*	100.00
2019SMG10	*Architeuthis dux*	99.41	KC701738.1	*Architeuthis dux*	100.00
2021SMG11	*Histioteuthis bonnellii*	99.84	NC_069187.1	*Histioteuthis bonnellii*	100.00
2021SMG12	*Haliphron atlanticus*	100.00	MN844883.1 / AY557516.1	*Haliphron atlanticus*	100.00
2021FAI13	*Haliphron atlanticus*	100.00	MN844883.1 / AY557516.1	*Haliphron atlanticus*	100.00
2021PIC14	*Haliphron atlanticus*	100.00%	MN844883.1 / AY557516.1	*Haliphron atlanticus*	100.00
2021PIC15	*Stigmatoteuthis arcturi*	98.70%	MG591287.1	*Stigmatoteuthis arcturi*	98.85
2021PIC15	*Stigmatoteuthis hoylei*	97.24%	AF000045.1	*Stigmatoteuthis hoylei*	97.21
2021SMG16	*Haliphron atlanticus*	99.84%	MN844883.1 / AY557516.1	*Haliphron atlanticus*	100.00
2021SMG17	*Haliphron atlanticus*	100.00	MN844883.1 / AY557516.1	*Haliphron atlanticus*	100.00
2021PIC18	*Haliphron atlanticus*	100.00	MN844883.1 / AY557516.1	*Haliphron atlanticus*	100.00
2021SMG19	*Haliphron atlanticus*	100.00	MN844883.1 / AY557516.1	*Haliphron atlanticus*	100.00
2021SMG20	*Haliphron atlanticus*	100.00	MN844883.1 / AY557516.1	*Haliphron atlanticus*	100.00
2021PIC21	*Haliphron atlanticus*	99.84	MN844883.1 / AY557516.1	*Haliphron atlanticus*	99.84
2021SMG22	*Haliphron atlanticus*	100.00	MN844883.1 / AY557516.1	*Haliphron atlanticus*	100.00
2021SMG23	*Haliphron atlanticus*	100.00	MN844883.1 / AY557516.1	*Haliphron atlanticus*	100.00
2021PIC24	*Haliphron atlanticus*	99.51	MN844883.1 / AY557516.1	*Haliphron atlanticus*	99.84
2021SMG25	*Haliphron atlanticus*	100.00	MN844883.1 / AY557516.1	*Haliphron atlanticus*	100.00
2021SMG26	*Haliphron atlanticus*	100.00	MN844883.1 / AY557516.1	*Haliphron atlanticus*	100.00
2021SMG27	*Haliphron atlanticus*	99.84	MN844883.1 / AY557516.1	*Haliphron atlanticus*	100.00
2021SMG28	*Haliphron atlanticus*	100.00	MN844883.1 / AY557516.1	*Haliphron atlanticus*	100.00
2021FAI29	*Haliphron atlanticus*	100.00	MN844883.1 / AY557516.1	*Haliphron atlanticus*	100.00
2021PIC30	*Haliphron atlanticus*	100.00	MN844883.1 / AY557516.1	*Haliphron atlanticus*	100.00
2021FAI31	*Haliphron atlanticus*	100.00	MN844883.1 / AY557516.1	*Haliphron atlanticus*	100.00
2021SMG32	*Haliphron atlanticus*	99.84	MN844883.1 / AY557516.1	*Haliphron atlanticus*	100.00
2021SMG33	*Haliphron atlanticus*	99.84	MN844883.1 / AY557516.1	*Haliphron atlanticus*	99.84
2021SMG34	*Haliphron atlanticus*	100.00	MN844883.1 / AY557516.1	*Haliphron atlanticus*	100.00
2021PIC35	*Haliphron atlanticus*	99.84	MN844883.1 / AY557516.1	*Haliphron atlanticus*	100.00
2021SMG36	*Haliphron atlanticus*	100.00	MN844883.1 / AY557516.1	*Haliphron atlanticus*	100.00
2021PIC37	*Haliphron atlanticus*	99.84	MN844883.1 / AY557516.1	*Haliphron atlanticus*	99.84
2021PIC38	*Haliphron atlanticus*	99.84	MN844883.1 / AY557516.1	*Haliphron atlanticus*	100.00
2021PIC39	*Haliphron atlanticus*	100.00	MN844883.1 / AY557516.1	*Haliphron atlanticus*	100.00
2021SMG40	*Haliphron atlanticus*	100.00	MN844883.1 / AY557516.1	*Haliphron atlanticus*	100.00
2021PIC41	*Haliphron atlanticus*	100.00	MN844883.1 / AY557516.1	*Haliphron atlanticus*	100.00
2022PIC42	*Haliphron atlanticus*	100.00	MN844883.1 / AY557516.1	*Haliphron atlanticus*	100.00
2022SMG43	*Haliphron atlanticus*	100.00	MN844883.1 / AY557516.1	*Haliphron atlanticus*	100.00
2022SMG44	*Haliphron atlanticus*	100.00	MN844883.1 / AY557516.1	*Haliphron atlanticus*	100.00
2022PIC45	*Haliphron atlanticus*	99.84	MN844883.1 / AY557516.1	*Haliphron atlanticus*	99.84
2022PIC46	*Haliphron atlanticus*	100.00	MN844883.1 / AY557516.1	*Haliphron atlanticus*	100.00
2022PIC47	*Ocythoe tuberculata*	99.84	AY557519.1	*Ocythoe tuberculata*	100.00
2022PIC48	*Histioteuthis reversa*	99.53	NC_069186.1	*Histioteuthis reversa*	99.84
2022PIC48				*Histioteuthis bonnellii*	99.84
2022SMG49	*Histioteuthis bonnellii*	99.37	NC_069187.1	*Histioteuthis bonnellii*	100.00
2022PIC50	*Haliphron atlanticus*	99.84	MN844883.1 / AY557516.1	*Haliphron atlanticus*	99.84
2022SMG51	*Haliphron atlanticus*	99.84	MN844883.1 / AY557516.1	*Haliphron atlanticus*	99.84
2022FAI53a	*Haliphron atlanticus*	99.84	MN844883.1 / AY557516.1	*Haliphron atlanticus*	99.84
2022PIC53b	*Haliphron atlanticus*	100.00	MN844883.1 / AY557516.1	*Haliphron atlanticus*	100.00
2022FAI54	*Haliphron atlanticus*	100.00	MN844883.1 / AY557516.1	*Haliphron atlanticus*	99.84
2022SMG55	*Haliphron atlanticus*	100.00	MN844883.1 / AY557516.1	*Haliphron atlanticus*	100.00
2022SMG56	*Haliphron atlanticus*	100.00	MN844883.1 / AY557516.1	*Haliphron atlanticus*	100.00
2022PIC57	*Haliphron atlanticus*	99.84	MN844883.1 / AY557516.1	*Haliphron atlanticus*	99.84
2022SMG58	*Haliphron atlanticus*	100.00	MN844883.1 / AY557516.1	*Haliphron atlanticus*	100.00
2022SMG59	*Haliphron atlanticus*	99.84	MN844883.1 / AY557516.1	*Haliphron atlanticus*	100.00
2022PIC60	*Haliphron atlanticus*	100.00	MN844883.1 / AY557516.1	*Haliphron atlanticus*	100.00
2022PIC61	*Haliphron atlanticus*	100.00	MN844883.1 / AY557516.1	*Haliphron atlanticus*	100.00
2022SMG62	*Haliphron atlanticus*	100.00	MN844883.1 / AY557516.1	*Haliphron atlanticus*	100.00
2022SMG63	*Haliphron atlanticus*	100.00	MN844883.1 / AY557516.1	*Haliphron atlanticus*	100.00
2022SMG64	*Histioteuthis bonnellii*	99.69	NC_069187.1	*Histioteuthis bonnellii*	100.00
2022SMG65	*Haliphron atlanticus*	100.00	MN844883.1 / AY557516.1	*Haliphron atlanticus*	100.00
2023PIC66	*Haliphron atlanticus*	100.00	MN844883.1 / AY557516.1	*Haliphron atlanticus*	100.00
2023PIC68	*Octopoteuthis sicula*	100.00	MT223203.1	*Octopoteuthis sicula*	100.00
2023PIC68	Taningia danae	99.84	EU735402.1		
2023PIC68	Octopoteuthis megaptera	99.84	EU735358.1		
2023SMG70	*Haliphron atlanticus*	99.38	MN844883.1 / AY557516.1	*Haliphron atlanticus*	100.00
2023SMG72	*Histioteuthis corona*	99.53	MK185932.1	*Histioteuthis corona*	99.84
2023PIC73	*Histioteuthis bonnellii*	99.69	NC_069187.1	*Histioteuthis bonnellii*	100.00
2023PIC77	*Haliphron atlanticus*	100.00	MN844883.1 / AY557516.1	*Haliphron atlanticus*	100.00
2023FAI79	*Taningia danae*	92.88	MG591434.1	no match	
2023PIC80	*Haliphron atlanticus*	99.69	MN844883.1 / AY557516.1	*Haliphron atlanticus*	100.00
2023PIC81	*Histioteuthis bonnellii*	100.00	MT223196.1	*Histioteuthis bonnellii*	100.00
2023PIC82	*Haliphron atlanticus*	100.00	MN844883.1 / AY557516.1	*Haliphron atlanticus*	100.00
2023PIC84	*Haliphron atlanticus*	99.85	MN844883.1 / AY557516.1	*Haliphron atlanticus*	99.84
2023PIC85	*Haliphron atlanticus*	99.85	MN844883.1 / AY557516.1	*Haliphron atlanticus*	99.84
2023PIC92	*Haliphron atlanticus*	100.00	MN844883.1 / AY557516.1	*Haliphron atlanticus*	100.00
2023PIC93	*Histioteuthis bonnellii*	99.84	MN844883.1 / AY557516.1	*Histioteuthis bonnellii*	99.84
2023FAI98	*Haliphron atlanticus*	100.0	MN844883.1 / AY557516.1	*Haliphron atlanticus*	100.00
2023FAI99	*Haliphron atlanticus*	100.00	MN844883.1 / AY557516.1	*Haliphron atlanticus*	100.00
2023FAI100	*Haliphron atlanticus*	100.00	MN844883.1 / AY557516.1	*Haliphron atlanticus*	100.00
2023SMG101	*Haliphron atlanticus*	99.69	MN844883.1 / AY557516.1	*Haliphron atlanticus*	99.69
2023PIC102	*Haliphron atlanticus*	99.85	MN844883.1 / AY557516.1	*Haliphron atlanticus*	99.84
2023PIC103	*Haliphron atlanticus*	99.85	MN844883.1 / AY557516.1	*Haliphron atlanticus*	99.84
2023PIC105	*Haliphron atlanticus*	100.00	MN844883.1 / AY557516.1	*Haliphron atlanticus*	100.00
2023PIC107	*Onykia carriboea*	99.68	MF171263.1	*Onykia carriboea*	99.84
2023PIC108	*Histioteuthis corona*	99.68	MG591290.1	*Histioteuthis corona*	99.84
2023PIC112	*Haliphron atlanticus*	99.85	MN844883.1 / AY557516.1	*Haliphron atlanticus*	99.84
2023PIC115	*Chiroteuthis veranii*	99.38	AY557529.1	*Chiroteuthis veranii*	99.84
2023SMG119	*Haliphron atlanticus*	100.00	MN844883.1 / AY557516.1	*Haliphron atlanticus*	100.00
2023SMG120	*Haliphron atlanticus*	99.85	MN844883.1 / AY557516.1	*Haliphron atlanticus*	99.84
2023SMG126	*Chiroteuthis veranii*	99.23	AY557529.1	*Chiroteuthis veranii*	99.84
2023PIC127	*Histioteuthis corona*	99.54	MK185932.1	*Histioteuthis corona*	99.84
2023SMG128	*Leachia atlantica*	99.84	MG591432.1	*Leachia atlantica*	99.31
2023FAI130	*Haliphron atlanticus*	100.00	MN844883.1 / AY557516.1	*Haliphron atlanticus*	100.00
2023FAI131	*Haliphron atlanticus*	100.00	MN844883.1 / AY557516.1	*Haliphron atlanticus*	100.00
2023FAI132	*Histioteuthis corona*	99.84	MK185932.1	*Histioteuthis corona*	100.00
2023FAI133	*Haliphron atlanticus*	100.00	MN844883.1 / AY557516.1	*Haliphron atlanticus*	100.00
2023PIC136	*Ommastrephes caroli*	100.00	MK995127.1	*Ommastrephes caroli*	100.00
2023PIC137	*Haliphron atlanticus*	100.00	MN844883.1 / AY557516.1	*Haliphron atlanticus*	100.00
2023FAI138	*Histioteuthis bonnellii*	100.00	NC_069187.1	*Histioteuthis bonnellii*	100.00
2024PIC140	*Haliphron atlanticus*	100.00	MN844883.1 / AY557516.1	*Haliphron atlanticus*	100.00
2024SMG141	*Haliphron atlanticus*	99.54	MN844883.1 / AY557516.1	*Haliphron atlanticus*	99.84
2024PIC142	*Haliphron atlanticus*	99.54	MN844883.1 / AY557516.1	*Haliphron atlanticus*	100.00
2024PIC144	*Haliphron atlanticus*	99.69	MN844883.1 / AY557516.1	*Haliphron atlanticus*	99.84
2024SMG145	*Taningia danae*	92.98	MG591434.1	no match	
2024SMG146	*Lepidoteuthis grimaldii*	99.84	KC860961.1	*Magnoteuthis magna*	100.00
2024SMG149	*Histioteuthis bonnellii*	92.68	AF000049.1	no match	
2024PIC162	*Histioteuthis bonnellii*	99.54	NC_069187.1	*Histioteuthis bonnellii*	100.00
2024PIC163	*Haliphron atlanticus*	99.84	MN844883.1 / AY557516.1	*Haliphron atlanticus*	99.84
2024PIC164	*Haliphron atlanticus*	99.39	MN844883.1 / AY557516.1	*Haliphron atlanticus*	99.85
2024PIC165	*Haliphron atlanticus*	100.00	MN844883.1 / AY557516.1	*Haliphron atlanticus*	100.00
2024PIC168	*Histioteuthis bonnellii*	100.00	MT223192.1	*Histioteuthis bonnellii*	100.00
2024PIC169	*Architeuthis dux*	99.85	KC701738.1	*Architeuthis dux*	100.00
2024SMG171	*Todarodes sagittatus*	99.52	MT223300.1	no match	
2024SMG172	*Todarodes sagittatus*	100.00	MT223313.1	no match	
2024SMG173	*Todarodes sagittatus*	100.00	MT223313.1	no match	
2024PIC179	*Haliphron atlanticus*	99.68	MN844883.1 / AY557516.1	*Haliphron atlanticus*	99.68
2024PIC185	*Architeuthis dux*	100.00	KC701738.1	*Architeuthis dux*	100.00

**Table 2.** List of taxa in the dataset and number of records (2019-2024).

Order	Family	Species	AphiaID	Nr. of records
Octopoda	Alloposidae	*Haliphron atlanticus* Steenstrup, 1857	341781	150
Oegopsida	Architeuthidae	*Architeuthis dux* Steenstrup, 1857	342218	3
	Chiroteuthidae	*Chiroteuthis veranii* (A. Férussac, 1835)	139125	2
	Cranchiidae	*Leachia atlantica* (Degner, 1925)	139425	1
	Histioteuthidae	*Histioteuthis bonnellii* (A. Férussac, 1834)	140111	9
		*Histioteuthis corona* (N. A. Voss & G. L. Voss, 1962)	140112	4
		*Histioteuthis reversa* (A. E. Verrill, 1880)	181381	1
		*Stigmatoteuthis arcturi* G. C. Robson, 1948 or *S. hoylei* (E. S. Goodrich, 1896)	410401 or 410403	1
	Lepidoteuthidae	*Lepidoteuthis grimaldii* Joubin, 1895	140193	1
	Mastigoteuthidae	*Magnoteuthis magna* (Joubin, 1913)	759131	1
	Octopoteuthidae	*Octopoteuthis sicula* Rüppell, 1844 ^(1)^	181379	1
		*Taningia danae* Joubin, 1931	140609	2
	Ocythoidae	*Ocythoe tuberculata* Rafinesque, 1814	140610	1
	Ommastrephidae	*Ommastrephes caroli* (Furtado, 1887) ^(2)^	342065	1
		*Todarodes sagittatus* (Lamarck, 1798)	140624	3
	Onychoteuthidae	*Onykia carriboea* Lesueur, 1821	140650	1

^(1)^ The BLAST on GenBank gave a 100% of identity for *Octopoteuthis sicula*. However, we cannot exclude the high similarity (99.84%) for *Taningia danae* or *Octopoteuthis megaptera*.

^(2)^ *Ommastrephes caroli* (Furtado, 1887), the neon flying squid, reported as *Ommastrephes bartramii* (Lesueur, 1821) in previous records for the region, is the North Atlantic species of the genus (Fernández-Álvarez et al. 2020).

**Table 3.** Teutophagous cetaceans presence associated with cephalopod samples collection. Gg: *Grampus griseus* (Risso’s dolphin), Gma: *Globicephala macrorhynchus* (short-finned pilot whale), Ha: *Hyperoodon ampullatus* (Northern bottlenose whale), Pm: *Physeter macrocephalus* (sperm whale), Ziphiidae (beaked whale): undetermined species.

**cephalopod species**	**Gg**	**Gma**	**Gme**	**Pm**	**Pm, Gg**	**Pm, Gg, Gma**	**Pm, Gma**	**Gg, Gma**	**Ha**	**Ziphi-idea**	**Other cetaceans ^(1)^**	**No cetaceans**	**N/A**	**TOTAL**
*Architeuthis dux*	1			2										3
*Chiroteuthis veranii*		1		1										2
*Haliphron atlanticus*	10	8	1	68	1	2	1	1	1	2	25|14	10	20	150
*Histioteuthis bonnellii*	1			4	1						3|0			9
*Histioteuthis corona*	1	1		2										4
*Histioteuthis reversa*				1										1
*Leachia atlantica*				1										1
*Lepidoteuthis grimaldii*				1										1
*Magnoteuthis magna*				1										1
*Octopoteuthis sicula*				1										1
*Ocythoe tuberculata*				1							0|1			1
*Ommastrephes caroli*												1		1
*Onykia carriboea*		1												1
*Stigmatoteuthis arcturi*				1										1
*Taningia danae*				1								1		2
*Todarodes sagittatus*		3												3
**Total**	**13**	**14**	**1**	**85**	**2**	**2**	**1**	**1**	**1**	**2**	**28|15**	**12**	**20**	**182**

^(1)^ Other cetaceans or non-teuthophagous cetaceans: baleen whales (blue whales, *Balænoptera musculus* and sei whale, *Balænoptera borealis*) and delphinidae (common dolphin, *Delphinus delphis*, Atlantic spotted dolphin, *Stenella frontalis*, bottlenose dolphin, *Tursiops truncatus*, striped dolphin, false killer whale, *Pseudorca crassidens* and killer whale, *Orcinus orca*), observed alone (28) or in association with teutophagous cetaceans (15) in the cephalopod sample area.
